# Spoken Word Learning in Children With Developmental Language Disorder or Dyslexia

**DOI:** 10.1044/2021_JSLHR-20-00217

**Published:** 2021-06-28

**Authors:** Suzanne M. Adlof, Lauren S. Baron, Bethany A. Bell, Joanna Scoggins

**Affiliations:** a University of South Carolina, Columbia

## Abstract

**Purpose:**

Word learning difficulties have been documented in multiple studies involving children with dyslexia and developmental language disorder (DLD; see also specific language impairment). However, no previous studies have directly contrasted word learning in these two frequently co-occurring disorders. We examined word learning in second-grade students with DLD-only and dyslexia-only as compared to each other, peers with both disorders (DLD + dyslexia), and peers with typical development. We hypothesized that children with dyslexia-only and DLD-only would show differences in word learning due to differences in their core language strengths and weaknesses.

**Method:**

Children (*N* = 244) were taught eight novel pseudowords paired with unfamiliar objects. The teaching script included multiple exposures to the phonological form, the pictured object, a verbal semantic description of the object, and spaced retrieval practice opportunities. Word learning was assessed immediately after instruction with tasks requiring recall or recognition of the phonological and semantic information.

**Results:**

Children with dyslexia-only performed significantly better on existing vocabulary measures than their peers with DLD-only. On experimental word learning measures, children in the dyslexia-only and DLD + dyslexia groups showed significantly poorer performance than typically developing children on all word learning tasks. Children with DLD-only differed significantly from the TD group on a single word learning task assessing verbal semantic recall.

**Conclusions:**

Overall, results indicated that children with dyslexia display broad word learning difficulties extending beyond the phonological domain; however, this contrasted with their relatively strong performance on measures of existing vocabulary knowledge. More research is needed to understand relations between word learning abilities and overall vocabulary knowledge and how to close vocabulary gaps for children with both disorders.

**Supplemental Material:**

https://doi.org/10.23641/asha.14832717

Vocabulary knowledge is critical for the acquisition of literacy skills (Perfetti, 2010; Quinn et al., 2015; Suggate et al., 2018), contributing to the development of both word-reading skills and overall reading comprehension. Children are more likely to correctly decode an unfamiliar printed word if that word already exists in their oral vocabulary (Duff & Hulme, 2012; Ricketts et al., 2007; Share, 1995). Understanding a text requires knowing meanings of most of the words within it (Nagy & Scott, 2000), and a meta-analysis of experimental studies indicates a causal relationship between vocabulary and reading comprehension (Elleman et al., 2009). As children develop proficiency in word reading, vocabulary and broader language skills play an increasingly important role in text comprehension (Foorman et al., 2018; Oslund et al., 2018). Unfortunately, many children with language-based learning difficulties, such as dyslexia and developmental language disorder (DLD), demonstrate difficulty learning new words (Alt et al., 2019; Kan & Windsor, 2010; Thomson & Goswami, 2010), and this reduced vocabulary, in turn, negatively impacts reading comprehension, academic progress, employment opportunities, and quality of life (Braze et al., 2007; Conti-Ramsden & Durkin, 2012; Johnson et al., 2010).

## Predictors of Word Learning

Learning a new spoken word requires constructing a phonological representation (i.e., a representation of how the word is pronounced), a semantic representation (i.e., the word's meaning), and links between the two (Gupta & Tisdale, 2009). These representations may be coarse initially and refined over time (Landauer et al., 2011; Metsala & Walley, 1998; Perfetti, 2007). Across the life span, most words are learned incidentally through communicative experiences. Infants and young children learn new words by extracting them from the ambient language environment (Ma et al., 2011). As such, the quantity and quality of spoken language to which children are exposed is a strong predictor of children's concurrent and future vocabulary size as well as academic outcomes (Goodman et al., 2008; Hart & Risley, 2003; Weisleder & Fernald, 2013).

However, language input is not the only factor that explains spoken word learning. Rather, several sources of evidence suggest that other child-level factors influence the degree to which children capitalize on word exposures. Longitudinal twin studies have reported significant genetic effects on late-talker status in toddlers (Zubrick et al., 2007) and vocabulary development between preschool and fourth grade (Byrne et al., 2009; Olson et al., 2011). Additionally, individual differences in word learning have been documented in experimental studies that have carefully controlled the quantity and quality of word exposures (Adlof & Patten, 2017; Gellert & Elbro, 2013; Language and Reading Research Consortium et al., 2016; Warmington et al., 2013).

Several factors have been proposed to explain individual differences in spoken word learning abilities. One set of theories emphasizes the influence of phonological processing (often measured using nonword repetition tasks) on new word learning. Numerous studies have demonstrated significant associations between nonword repetition abilities, word learning abilities, and overall vocabulary size (Adlof & Patten, 2017; Gathercole et al., 1999; Gupta, 2003; Hoff et al., 2008; Melby-Lervåg et al., 2012). These findings have been interpreted as evidence for phonological constraints on new word learning, such that children with phonological deficits may have difficulty constructing precise form representations or links between form and meaning representations (Abel & Schuele, 2014).

Other research has implicated semantic processing and the robustness of existing semantic networks in new word learning. The size of an individual's existing vocabulary predicts their ability to learn new words (Adlof & Patten, 2017; Cain et al., 2004; Ewers & Bronson, 1999). Additionally, studies of infant word learning indicate that the structure of a child's existing semantic network predicts which new words will enter the child's vocabulary (Hills et al., 2009; see also Storkel & Adlof, 2009). Further evidence suggests that children leverage existing categorical knowledge during word learning, such that newly learned words from densely populated categories are recognized more efficiently than words from sparsely populated categories (Borovsky et al., 2016). This study examined phonological and semantic aspects of word learning in children with disorders characterized by strengths and weaknesses in these areas.

## Word Learning Difficulties in Children With DLD and Dyslexia

Word learning difficulties have been associated with many types of reading and language difficulties, including DLD and dyslexia. DLD and dyslexia are separate disorders that frequently co-occur (Adlof et al., 2017; Bishop et al., 2009; Catts et al., 2005; Eisenmajer et al., 2005; Ramus et al., 2013). DLD is a significant impairment in the ability to understand and produce spoken language despite otherwise apparently typical development (Bishop et al., 2017). Prior to CATALISE (Bishop et al., 2016), the term *specific language impairment* (SLI) was commonly used to refer to children with unexplained language impairment. SLI can be viewed as a major subgroup of the full population of DLD (e.g., Norbury et al., 2016), with DLD including children with a range of nonverbal cognitive abilities but excluding intellectual disabilities, and SLI setting a minimum criterion for nonverbal intelligence scores, often 1 *SD* below the mean. The language deficits of children with DLD are heterogeneous. As a group, children with DLD display marked difficulties with semantic and syntactic processing, as well as discourse-level language skills. Numerous studies have documented word learning difficulties in children with DLD, with most studies prior to 2015 focusing on children whose nonverbal intelligence scores were no more than 1 *SD* below the population mean, thus meeting customary criteria for SLI (cf. Jackson et al., 2019; Kan & Windsor, 2010). Kan and Windsor's (2010) meta-analysis of 28 studies found that these children scored, on average, 0.6 *SD*s lower than age-matched peers on word learning measures, with significant group differences observed when learning was measured with both production (*g* = 0.40) and comprehension (*g* = 0.72) tasks.

The word learning difficulties of children with DLD have been linked to both phonological and semantic deficits; however, this research has not considered the potential comorbidity of dyslexia and DLD. For example, children with DLD differed from typically developing peers in nonword repetition and vocabulary size (Estes et al., 2007; Rice & Hoffman, 2015), and both nonword repetition and existing vocabulary knowledge predicted their ability to learn new words in training studies (Gray, 2004). Children with DLD have also displayed difficulty with learning both phonological and semantic aspects of new words (Alt et al., 2004; Nash & Donaldson, 2005). In addition to linguistic factors, nonverbal cognitive abilities have been found to moderate word learning in children with DLD, even when inclusionary criteria required all children to score within normal limits on measures of nonverbal intelligence (Kan & Windsor, 2010).

Dyslexia is defined as a specific impairment of word reading most commonly attributed to a biologically based core deficit in phonological processing (Lyon et al., 2003; Vellutino et al., 2004). This phonological impairment leads to difficulty learning sound–symbol correspondences and manifests as difficulty with decoding and spelling words (Vellutino et al., 2004). Children with dyslexia have also been shown to have difficulty on tasks of nonword repetition (Catts et al., 2005) and spoken word learning (Alt et al., 2017, 2019; Thomson & Goswami, 2010). Poor vocabulary has been implicated in young children with or at risk for dyslexia (Vellutino et al., 2004). However, some studies suggest vocabulary can be a mitigating factor in adult readers with dyslexia (Cavalli et al., 2016; Wiseheart & Altmann, 2018).

In comparison to studies of children with DLD, studies of word learning in children with dyslexia have primarily focused on phonological aspects rather than semantic. For example, when the required response modality is verbal (i.e., producing a spoken word), children with dyslexia have significant difficulty pairing verbal stimuli and nonverbal referents compared to age- and reading-matched peers (Kalashnikova & Burnham, 2016; Kamhi et al., 1990; Litt & Nation, 2014; Mayringer & Wimmer, 2000; Messbauer & de Jong, 2003). Alternatively, in tasks where the required response modality is nonverbal (i.e., pointing or selecting), children with dyslexia were able to pair a verbal stimulus with its nonverbal referent as accurately as their peers with typical reading development (Baron et al., 2018; Litt & Nation, 2014). Such results have been interpreted as evidence that phonological deficits impede word learning in children with dyslexia. Additionally, there is evidence that these phonological or verbal learning deficits are a key contributor to individual differences in reading ability (Clayton et al., 2018).

Whereas phonological processing deficits are considered core deficits in dyslexia (Lyon et al., 2003), the status of phonological skills in children with DLD is less clear. Most studies of each disorder have relied on convenience samples, which likely included many children with comorbid DLD and dyslexia (DLD + dyslexia). For example, samples recruited from clinical caseloads have suggested higher rates of comorbidity than population-based samples (Catts et al., 2005; McArthur et al., 2000). In studies that do not account for comorbidities, the conclusions about characteristics of children with one disorder can be influenced by the presence of individuals in the sample with the other disorder. In studies that have controlled for the comorbidity between language and reading impairment, some children with DLD had average word reading skills (DLD-only) and their phonological processing skills did not significantly differ from typically developing peers (Catts et al., 2005; Eisenmajer et al., 2005). Other studies found that children with DLD-only still exhibited poorer phonological processing than typically developing (TD) controls, but they also scored significantly better than children with DLD + dyslexia (De Groot et al., 2015; Ramus et al., 2013).

With some exceptions (e.g., Alt et al., 2017, 2019; Kamhi et al., 1990; Malins et al., 2020), the comorbidity between DLD and dyslexia has been largely ignored in word learning studies, which complicates the understanding of word learning difficulties in each disorder. To our knowledge, there are only two prior studies that have examined the word learning abilities of children with DLD + dyslexia as compared to children with dyslexia with average language skills (dyslexia-only) and TD children (Alt et al., 2019; Malins et al., 2020). Alt et al. (2019) taught second-grade children with DLD + dyslexia, dyslexia-only, and TD the pseudoword names and semantic features of novel objects in six games (i.e., conditions) that manipulated phonological or visuospatial features such as pseudoword length or visual similarity of the novel object referents. Each game comprised the same five tasks that varied in phonological and visual-semantic demands. Children with DLD + dyslexia demonstrated lower accuracy than the dyslexia-only and TD groups across a majority of games/tasks requiring either phonological or semantic processing. In contrast, children with dyslexia-only primarily struggled with games/tasks requiring phonological processing. Thus, Alt et al.'s (2019) findings provided evidence for differences in the word learning difficulties of children with dyslexia with and without DLD. Children with dyslexia-only were primarily affected in the phonological domain of word learning, whereas children with DLD + dyslexia showed broader and more severe deficits. Similarly, Malin et al. (2020) concluded that children with DLD + dyslexia demonstrated broad word learning impairments—both during learning and 1 day later—compared to peers with dyslexia-only. However, the status of word learning in children with DLD-only is as yet unknown.

## Study Purpose

The majority of existing word learning studies have not accounted for the high comorbidity of DLD and dyslexia, which precludes accurate description of word learning profiles for either disorder. Thus, the purpose of this study was to examine group differences in global measures of word learning in children with DLD-only and dyslexia-only as compared to each other, to peers with DLD + dyslexia, and to peers with TD. We hypothesized that children with dyslexia-only would display difficulty learning new phonological forms. This hypothesis was based on the assumption of core deficits in phonological processing in children with dyslexia (Lyon et al., 2003). In contrast, we hypothesized that children with DLD-only would display a primary difficulty with word learning tasks that require more semantic knowledge, given their broader linguistic deficits (Leonard, 2014). Finally, we predicted that children with DLD + dyslexia would show greater and more distributed word learning difficulties than either group of children with a single deficit.

The analyses presented in this article focus on DLD and dyslexia groups defined by their primary inclusionary criterion—that is, language or word reading skills below normal limits. However, we also conducted analyses that employed a nonverbal IQ cutoff for all participants, excluding those who scored more than 1 *SD* below the mean, as has been common in studies of SLI (Kan & Windsor, 2010; Leonard, 2014) and dyslexia (Catts et al., 2005; Mayringer & Wimmer, 2000; but see Stuebing et al., 2009). As similar results were obtained across the two classification methods, the analyses with the nonverbal IQ cutoff are presented in the Supplemental Material S1.

## Method

Participants included 244 children, aged 7 years 10 months to 9 years 4 months (*M*_age_ = 95.75 months or nearly 8 years). Children were recruited during second grade from a large, suburban school district in South Carolina across three academic years. The general procedure began with classroom screenings of language and reading ability (Adlof et al., 2017) followed by assessment of individual child participants. All study procedures were approved by the University of South Carolina Institutional Review Board.

Parents provided informed consent and completed a brief questionnaire requesting demographic information, language background, and information about their child's medical and educational history. Based on parent report, all participants spoke English as their first and only language, had no hearing impairment, uncorrected vision impairment, or other medical or developmental history that would interfere with speech or language development. Child participants completed a battery of norm-referenced language and reading assessments as well as the experimental word learning protocol. Data collection sessions were video- and audio-recorded for off-line scoring and reliability checking.

### Norm-Referenced Measures

#### Subgrouping Assessments

Language ability was measured using the Core Language Score from the Comprehensive Evaluation of Language Fundamentals–Fourth Edition (CELF-4; Semel et al., 2003), which is a composite of scores from four subtests assessing different aspects of language comprehension and production. For children between 5 and 8 years old, the four core language subtests are Concepts and Following Directions, which measures a child's ability to follow verbal instructions of increasing length and complexity; Word Structure, a measure of morphology and pronoun use; Recalling Sentences, a measure of sentence repetition; and Formulated Sentences, a measure of a child's ability to generate a spoken sentence using a word provided by the examiner. For children between 9 and 12 years old, the Word Structure subtest was replaced with Word Classes, a measure of a child's ability to understand and explain semantic relationships between words. Children were classified as having DLD if they scored ≤ the 16th percentile (SS ≤ 85) on the Core Language composite of the CELF-4. According to the test manual, this cut score provides 100% sensitivity and 82% specificity for identifying children with language impairment.

This study was conducted in a region of the country where many people speak a dialect that varies from mainstream American English (MAE) such as African American English or Southern White English. Therefore, it was important to ensure that children with typical language abilities who spoke a nonmainstream dialect were not misclassified as having DLD (cf. Hendricks & Adlof, 2017). We used the Diagnostic Evaluation of Language Variance—Screening Test (DELV-ST; Seymour et al., 2003) as part of a two-step checking procedure to verify the language impairment status of children whose dialect varied from the mainstream. Specifically, in addition to meeting the CELF-4 Core Language Score cutoff (SS ≤ 85), children who were classified by the DELV-ST as exhibiting “some” or “strong” variation from MAE had to be classified by the DELV-ST as having “medium–high” to “highest” risk of language impairment in order to remain in the DLD group for analysis. Children who scored below the CELF-4 cutoff that were classified as having some or strong variation from MAE and for whom the DELV-ST risk status suggested lowest or low–medium risk for language impairment were excluded from study analyses.

Word reading was measured using the Basic Skills Cluster of the Woodcock Reading Mastery Test–Third Edition (WRMT-3; Woodcock, 2011), which is a composite score derived from the Word Identification and Word Attack subtests. For each subtest, children read a list of increasingly challenging single words (Word Identification) or pseudowords (Word Attack). Children were classified as having dyslexia if they scored ≤ the 16th percentile (SS ≤ 85) on the Basic Skills Cluster of the WRMT-3. Cut scores to ensure inclusion of children with dyslexia vary widely across studies, ranging from the 7th percentile (Badian et al., 1990) to the 30th percentile (Manis et al., 1996); our selected cutoff represents a midpoint and is consistent with studies that have used the same test (Catts et al., 2005; Joanisse et al., 2000; Siegel, 2008).

Using these criteria, participants were classified into four subgroups. Children were classified as having DLD + dyslexia if they met the above criteria for both disorders. To avoid borderline cases, children in the DLD-only and dyslexia-only subgroups were required to have scores > the 25th percentile (SS > 90) for their nonimpaired skill (cf. Catts et al., 2006). Children were classified as TD if they scored > 25th percentile and < 85th percentile (SS > 90 and < 116) on both measures. Similar to prior research with these subgroups (Adlof & Catts, 2015; Catts et al., 2006), this criterion excludes very high performers, helps ensure that participants with TD have “average” reading and language skills, and leads to a more conservative comparison between typical and impaired groups. See Table 1 for descriptive statistics and group comparisons on the subgrouping assessments.

**Table 1.** Descriptive statistics and group comparisons for subgrouping and descriptive assessments.

	Group scores				ANOVA results				
Subgrouping assessment	TD (*n* = 90)	DLD (*n* = 53)	DYS (*n* = 32)	DLD + DYS (*n* = 69)	*df (3,*	*F*	*p*	*R* ^2^	Group comparison
CELF-4 *M* (*SD*)	100.96 (6.84)	79.49 (5.27)	97.31 (5.53)	73.84 (8.76)	240	241.47	< .001	.751	TD > DYS > DLD > DLD + DYS
WRMT-3 *M* (*SD*)	101.24 (6.17)	101.13 (7.47)	80.22 (3.62)	74.70 (7.37)	240	284.52	< .001	.781	TD = DLD > DYS > DLD + DYS
**Descriptive assessment**	**TD** ^**a**^	**DLD** ^**b**^	**DYS**	**DLD** + **DYS** ^**c**^	***df (3,***	***F***	***p***	***R*** ^**2**^	**Group comparison**
TOWRE-2 *M* (*SD*)	102.11 (9.79)	101.13 (9.47)	79.12 (8.02)	75.35 (12.17)	239	119.80	< .001	.601	TD = DLD > DYS = DLD + DYS
CTOPP-2 *M* (*SD*)	92.89 (11.63)	83.70 (10.68)	88.09 (12.19)	75.96 (9.58)	239	31.88	< .001	.286	TD = DYS, DYS = DLD, TD > DLD > DLD + DYS, DYS > DLD + DYS
PPVT-4 *M* (*SD*)	107.11 (10.91)	93.72 (7.97)	104.84 (8.69)	91.41 (9.40)	238	44.27	< .001	.358	TD = DYS > DLD = DLD + DYS
EVT-2 *M* (*SD*)	105.90 (8.63)	93.45 (7.08)	101.37 (6.29)	87.32 (8.31)	239	78.82	< .001	.494	TD > DYS > DLD > DLD + DYS
TONI-4 *M* (*SD*)	106.73 (8.68)	101.44 (9.40)	100.91 (9.31)	95.24 (8.32)	234	21.56	< .001	.217	TD > DLD = DYS > DLD + DYS

*Note.* A series of one-way ANOVAs were estimated to examine group differences on the subgrouping and descriptive assessments. Pairwise comparisons using Tukey's honestly significant difference test were applied to maintain an experiment-wise Type I error rate of .05 for each model. ANOVA = analysis of variance; TD = typical development; DLD = developmental language disorder; DYS = dyslexia; CELF-4 = Comprehensive Evaluation of Language Fundamentals–Fourth Edition; WRMT-3 = Woodcock Reading Mastery Test–Third Edition; TOWRE-2 = Test of Word Reading Efficiency–Second Edition; CTOPP-2 = Comprehensive Test of Phonological Processing–Second Edition; PPVT-4 = Peabody Picture Vocabulary Test–Fourth Edition; EVT-2 = Expressive Vocabulary Test–Second Edition; TONI-4 = Test of Nonverbal Intelligence–Fourth Edition.

a Sample sizes differed on the PPVT-4 (*n* = 89) and TONI-4 (*n* = 86).

b Sample sizes differed on the TOWRE-2 and TONI-4 (*n* = 52).

c Sample sizes differed on the CTOPP-2, PPVT-4, EVT-2, and TONI-4 (*n* = 68).

#### Descriptive Assessments

In addition to the subgrouping assessments, we administered several other norm-referenced assessments to further describe the language and reading profiles of each group. Word reading fluency was measured using the Test of Word Reading Efficiency–Second Edition (Torgesen et al., 2012). This test consists of a Sight Word Efficiency subtest and a Phonemic Decoding subtest, which require speeded reading of real words and pseudowords, respectively. Phonological memory was measured using the Nonword Repetition and Memory for Digits subtests of the Comprehensive Test of Phonological Processing–Second Edition (CTOPP-2; Wagner et al., 2013). Scores on these subtests can be combined to derive a Phonological Working Memory Composite score. Receptive vocabulary knowledge was measured using the Peabody Picture Vocabulary Test–Fourth Edition (Dunn & Dunn, 2007), and expressive vocabulary knowledge was measured using the Expressive Vocabulary Test–Second Edition (Williams, 2007). Finally, nonverbal cognitive skills were assessed with the Test of Nonverbal Intelligence–Fourth Edition (TONI-4; Brown et al., 2010). See Table 1 for descriptive statistics and group comparisons on the descriptive assessments.

Note that the pattern of performance on these descriptive assessments generally mirrored that of the subgrouping assessments. Both groups of children with dyslexia (dyslexia-only and DLD + dyslexia) showed significantly poorer performance than children with DLD-only and TD on measures of word reading fluency, and both groups of children with DLD (DLD-only and DLD + dyslexia) showed significantly poorer performance than children with dyslexia-only and TD on measures of existing vocabulary knowledge. On average, phonological memory was relatively poor for all impairment subgroups when compared to children with TD. Finally, although the majority of children in all groups obtained typical nonverbal IQ scores, the TD group obtained higher scores than the DLD-only and dyslexia-only groups, who in turn obtained higher scores than the DLD + dyslexia group. We also analyzed our data using more restrictive SLI criteria in which we excluded all children with SS < 85 on the TONI-4. Descriptive data and results for these participants are presented in the Supplemental Material S1.

### Word Learning Protocol

Word learning abilities were assessed with computerized training and testing tasks modeled after Adlof and Patten (2017). Children were taught eight pairs of novel names and unfamiliar object referents (see Table 2). The novel word stimuli consisted of eight, two-syllable consonant–vowel–consonant–vowel–consonant–vowel– pseudowords selected from the Irvine Phonotactic Online Dictionary (Vaden et al., 2009), which had medium phonotactic probability and neighborhood density relative to all two-syllable real words within the database. All items had three phonological neighbors, biphone probability ranging from .003 to .005, and positional segment probability ranging from .048 to .057.

**Table 2.** Word learning stimulus descriptions.

Novel word	Real object referent	Category	Feature 1 (visible)	Feature 2 (visible)	Feature 3 (invisible)	Feature 4 (invisible)
/felɪdʒ/	Helmetted Hornbill	Bird	A /felɪdʒ/ has wrinkled brown skin on its neck.	It has a large bony plate on its beak.	A /felɪdʒ/ eats bugs.	A /felɪdʒ/ likes warm weather.
/gɛθəl/	Secretary bird	Bird	A /gɛθəl/has black and white feathers on its wings.	It has a small patch of feathers on its head.	A /gɛθəl/eats fish.	A /gɛθəl/ likes cool weather.
/ʃobət/	Horned Melon	Fruit	A /ʃobət/ has spikey yellow skin.	It has lumpy green pulp on the inside.	A /ʃobət/ grows on vines.	A /ʃobət/ tastes sour.
/tɛpɪk/	Rambutan	Fruit	A /tɛpɪk/ has hairy red skin.	It has smooth white pulp on the inside.	A /tɛpɪk/ grows on trees.	A /tɛpɪk/ tastes sweet.
/limɪdʒ/	Belay device	Tool	A /limɪdʒ/ has two small loops on the handle.	It has folding hinges on top.	A /limɪdʒ/ is light-weight.	A /limɪdʒ/ is used for climbing trees.
/mɪʒɪk/	Knurling tool	Tool	A /mɪʒɪk/ has two grooved wheels on the ends.	It has a large knob on top.	A /mɪʒɪk/ is very heavy.	A /mɪʒɪk/is used for stamping things.
/kɑzət/	Aptera Car	Vehicle	A /kɑzət/ has three small wheels.	It has covered seats for riders.	A /kɑzət/ runs on electricity.	A /kɑzət/ travels at fast speeds.
/daʊfəl/	Concept bike	Vehicle	A /daʊfəl/ has one large wheel.	It has one seat that is not covered.	A /daʊfəl/ runs on gasoline.	A /daʊfəl/ is only driven by police.

The eight unfamiliar object referents came from four familiar categories (i.e., birds, fruits, tools, and vehicles). Photographs of the referents were obtained from stock photo websites and Google Images and modified using Adobe Photoshop such that they were all of similar size and displayed on a white background. A separate pilot study was conducted to confirm that the referents were unfamiliar to children and that children could determine category membership of each referent. Each referent had two visible features and two invisible features. For example, yellow skin and green pulp were named as the visible features for one of the fruits, whereas its invisible features were that it grows on vines and tastes sour. The invisible features were plausible, but not necessarily true descriptions of the real object referents. By including two items per category, the invisible features were less likely to be inferred from category membership alone.

#### Training

Children were instructed that they would be helping an astronaut learn about life on an alien planet before she traveled there. For each object referent, a recorded script introduced the corresponding name, category, and four semantic features as well as provided opportunities to practice saying the name (phonological recall) and find the object within an array of all eight object referents (semantic recognition). Recorded feedback on the accuracy of naming and finding responses was provided during the training. These script elements were repeated in three separate teaching blocks, and the order of the script elements was varied within and across the blocks (see Supplemental Material S2). This variation was intended to promote engagement and facilitate word learning based on evidence that spaced retrieval improves learning (Roediger & Butler, 2011; see also Haebig et al., 2019; Leonard et al., 2019). Within each teaching block, the order of presentation of the target words was randomized. During the complete training session, children received 22 exposures to the spoken word form, three opportunities to practice naming each object with feedback, and three opportunities to identify the correct object within an array including all eight object referents with feedback.

#### Word Learning Assessment

Five tasks were designed to assess children's recall and recognition of phonological and semantic information provided during training (see Table 3). We classified the assessment as phonological or semantic based on the kind of information that children had to supply in their responses (Nash & Donaldson, 2005), but it is important to note that all five of the tasks also measured links between phonology and semantics. Tasks that required spoken production of the pseudoword were considered phonological while tasks that required visual or spoken description of the referent were considered semantic. The order of measures was fixed, as follows, but the order of items within each measure was randomized across participants. Participants did not receive any feedback regarding the accuracy of their responses during the assessment tasks. Tasks that required a spoken response were audio-recorded for off-line transcription and scoring. For each task, the primary outcome was an accuracy score, which required correct phonology, semantics, and links between them. Secondary scores were also derived for four tasks in order to gain more information about participants' phonological or semantic learning from inaccurate responses. Specifically, we derived secondary scores for the three recall tasks in order to examine participants' recall of phonological or semantic information that was not correctly linked. We also compared the frequency of phonological, semantic, and other errors for each group within the listening recognition task.

**Table 3.** Summary of word learning assessment tasks and score types.

Task	Measurement	Instructions to child	Score type	Score description	Correct link required?
Naming	Phonological Recall	[object displayed on screen] “Tell me what this is called.”	**Naming accuracy**	**No. of objects correctly named**	**Yes**
			Naming recall	Total no. of pseudowords recalled	No
			Naming difference	= Naming recall – naming accuracy; represents no. of pseudowords recalled with incorrect links to semantics	No
Listening	Phonological Recognition	“.… Listen carefully and tell me which one is the right name of the picture.” [object displayed on screen; four words played]	**Listening accuracy**	**No. of objects correctly named**	**Yes**
			Phonological error	Selection of the mispronounced pseudoword	Yes
			Semantic error	Selection of correctly pronounced semantic foil	No
			Combined error	Selection of the mispronounced semantic foil	No
Drawing	Semantic Recall (Nonverbal)	“Draw a [pseudoword].”	**Drawing accuracy**	**No. of objects correctly drawn**	**Yes**
			Drawing recall	Total no. of objects recalled	No
			Drawing difference	= Drawing recall – drawing accuracy; represents no. of objects recalled with incorrect links to pseudowords	No
Describing	Semantic Recall (Verbal)	“Tell me everything you know about a [pseudoword].”	**Describing accuracy**	**No. of categories and features correctly recalled**	**Yes**
			Describing recall	Total no. of correct categories and features recalled	No
			Describing difference	= Describing recall – describing accuracy; represents no. of categories and features recalled with incorrect links to pseudowords	No
Finding	Semantic Recognition	[8 objects displayed on screen.] “Find the [pseudoword].”	**Finding accuracy**	**No. of objects correctly identified**	**Yes**

*Note.* Accuracy scores used in primary analyses are bolded. All scores for the naming, listening, drawing, and finding tasks had a maximum possible value of 8. The describing task all had a maximum possible value of 40.

The naming task assessed phonological recall. Participants were shown a picture of a novel object and asked to recall its name. The primary outcome was the naming accuracy score, which represented the number of objects correctly named based on a dichotomous rating of correct or incorrect for each word. In addition, we also calculated a naming recall score, which is a more inclusive measure of phonological learning representing the number of unique names (i.e., pseudowords) that were recalled, regardless of whether they were linked to the correct object. The naming accuracy and naming recall scores each had a maximum value of 8. To examine whether groups differed in their recall of names overall, or of names that were correctly linked to their semantic referent, we calculated a naming difference score by subtracting the naming accuracy score from the naming recall score.

The listening task assessed phonological recognition. Participants were shown a picture and heard the following recorded prompt: “I will show you a picture, and you will hear four words. Listen carefully and tell me which one is the right name of the picture.” Four recorded pseudowords were played, including the correctly pronounced target word, a phonological foil, a semantic foil, and a combined phonological/semantic foil. The phonological foil was a mispronunciation of the target word that differed by one phoneme from the correct pronunciation (e.g., /tɛtɪk/ for /tɛpɪk/). The semantic foil was a correctly pronounced word from the same category as the target (e.g., /ʃobət/). The combined foil was a mispronunciation of the semantic foil (e.g., /ʃodət/). Thus, for each item, participants heard two correctly pronounced words and two mispronounced words. The order of presentation of the correct answer was randomized across trials. After the four options were played, children were asked to say the correct word for the displayed object. The primary outcome was the listening accuracy score, which represented the number of objects correctly named within the listening task. Incorrect responses were also classified according to foil type, that is, as phonological, semantic, combined, or other errors (i.e., giving a different response from the four listening options). With a total of eight items, the maximum value for listening accuracy score and for all error types was 8, and the sum of the listening accuracy score and all error types could not exceed 8. For example, a child could earn a listening accuracy score of 6 and make 1 phonological error and 1 semantic error.

The drawing task assessed nonverbal semantic recall. Children heard a recorded pseudoword and were asked to draw a picture of the corresponding object. The primary outcome was the drawing accuracy score, which represented the number of items for which the drawing could be clearly identified as the target object (max value = 8). We also calculated a drawing recall score, a more inclusive measure of semantic learning that reflected the number of unique novel objects that were recalled within the task, regardless of whether they were linked with the correct word (max value = 8). The drawing difference score was derived by subtracting the drawing accuracy score from the drawing recall score. This difference score was used to examine whether groups differed in their recall of objects overall, or of objects that were linked to the correct pseudoword.

The describing task assessed verbal semantic recall. Children were asked to “tell me everything you know about a [word].” The primary outcome was the describing accuracy score, which was calculated by summing the number of correctly recalled categories and features across all words (one category and four features per word). Each item could receive up to 5 points; thus, total score across all eight items ranged from 0 to 40. We also calculated a describing recall score, a more inclusive measure of semantic learning that reflected the sum of the number of categories and features recalled within the task without the constraint of accurate linking (max value = 40). The describing difference score was derived by subtracting the describing accuracy score from the describing recall score. This difference score was used to examine whether groups differed in their recall of semantic features overall, or of features that were linked to the correct pseudoword.

The finding task assessed semantic recognition. On each trial, children saw an array with pictures of all eight objects and were asked, “Can you find the [word]?” Participants pointed to an object on the screen, and the assessor pressed the corresponding number on a keypad. The primary outcome was the finding accuracy score, which represented the number of items correctly identified. This score was calculated by the computer and had a maximum value of 8.

In summary, the primary outcome for each task was an accuracy score that required correct links between newly learned phonological and semantic representations. The maximum score for each primary outcome was 8, except for the describing task where participants could earn a score of 40. Secondary scores were calculated to gather additional information about participants' phonological and/or semantic learning through their error responses.

### Reliability

Data were collected across three academic years. In Year 1, all measures were double scored to ensure reliability, and disagreements were reconciled through discussion with the lead assessor and/or principal investigator. In Years 2 and 3, scorers had to participate in training and achieve 95% or better agreement on an item-by-item analysis for at least five sample participants on each assessment. For assessments requiring whole-word transcription, scorers were allowed no more than five transcription differences with a trained scorer across five sample participants.

Reliability was assessed for each assessment by double scoring a random sample of at least 20% of each scorer's work. Reliability scorers used blank protocols and audio/video recordings of the assessment administration, so they were blinded to the initial score. For Years 2 and 3 combined, reliability of norm-referenced assessments was 93.5% for CELF-4, 92.2% for DELV-ST, 96.8% for WRMT-3, 97.0% for Test of Word Reading Efficiency–Second Edition, 96.8% for CTOPP, 99.5% for Expressive Vocabulary Test, 99.8% for Peabody Picture Vocabulary Test, and 95.7% for TONI-4. For word learning assessments, reliability of the primary outcome scores was 97.3% for naming accuracy, 92.0% for listening accuracy, 91.8% for drawing accuracy, and 91.1% for describing accuracy. The finding accuracy score was generated by the computer program and did not require reliability assessment.

### Data Analysis

We used multiple analytic procedures to answer our research questions. First, a series of analyses of variance (ANOVAs) were estimated to examine group differences on the primary accuracy score from each of the five word learning tasks—naming, listening, drawing, describing, and finding—with planned pairwise comparisons using Tukey's honestly significant difference (HSD) to maintain an experiment-wise Type I error rate of .05 for each model. All assumptions were examined, and only one violation was noted: There were unequal group variances for the describing model. To account for this violation, we interpreted Welch's ANOVA. For effect size, we report *R* ^2^ for the overall ANOVA and Hedge's *g* for pairwise comparisons. Hedge's *g* is interpreted similarly to Cohen's *d* but weights effects according to the relative size of each sample. We interpret *g* values up to 0.4 as small, up to 0.7 as medium, and values of 0.8 and above as large (Cohen, 1988; Fritz et al., 2012).

We also conducted a series of secondary analyses using the additional scoring method data. For the naming, drawing, and describing tasks, recall scores allowed us to better characterize the nature of word learning strengths and weaknesses by considering general learning of phonological or semantic information without the constraint of accurate linking. Difference scores provide a measurement of phonological or semantic information that was learned but inaccurately linked. We estimated a series of ANOVAs to examine difference scores across subgroups, again interpreting Welch's ANOVA when appropriate and using Tukey's HSD test for pairwise comparisons. For the listening task, we present the proportion of responses by error type and group, and we provide descriptive interpretation.

## Results

### Group Differences in Word Learning Accuracy Scores

Statistically significant between-group differences were found for the primary accuracy score on all five tasks; see Table 4 for detailed ANOVA results and Table 5 for descriptive data, effect sizes, and Tukey's HSD test results. In the naming task, which measured phonological recall, the TD group obtained significantly higher naming accuracy scores than the dyslexia-only (*g* = 0.70) and DLD + dyslexia (*g* = 0.56) groups. No other group differences were statistically significant (all other effect sizes were small to moderate, *g* < 0.43).

**Table 4.** One-way ANOVA summary table examining group differences on the primary outcome for each word learning task.

Source	Sum of squares	*df*	Mean square	*F*	*p* value	*R* ^2^
**Naming**						
Group	28.28	3	9.43	6.51	.0003	.075
Error	347.67	240	1.45			
**Listening**						
Group	179.92	3	59.97	20.87	< .0001	.207
Error	689.63	240	2.87			
**Drawing**						
Group	178.74	3	59.58	16.74	< .0001	.173
Error	854.03	240	3.56			
**Describing** ^a^						
Group	843.51	3	281.17	11.36	< .0001	.140
Error	5190.47	107.2	21.63			
**Finding**						
Group	156.48	3	52.16	11.45	< .0001	.125
Error	1249.52	240	4.55			

a Results are from Welch's ANOVA to account for unequal group variances. ANOVA = analysis of variance.

**Table 5.** Primary accuracy scores of word learning tasks by group, effect size, and significance of pairwise comparisons.

	Group scores				Effect size (Hedge's *g*) and significance of pairwise comparisons					
Task	TD (*n* = 90)	DLD (*n* = 53)	DYS (*n* = 32)	DLD + DYS (*n* = 69)	TD vs. DLD	TD vs. DYS	TD vs. DLD + DYS	DLD vs. DYS	DLD vs. DLD + DYS	DYS vs. DLD + DYS
Naming *M* (*SD*)	1.59 (1.35)	1.21 (1.32)	0.69 (0.74)	0.90 (1.07)	0.284	0.737*	0.558*	0.457	0.262	−0.215
Listening *M* (*SD*)	5.56 (1.59)	4.83 (1.87)	4.09 (1.45)	3.48 (1.80)	0.430	0.945*	1.235*	0.429	0.737*	0.359
Drawing *M* (*SD*)	4.30 (1.96)	3.53 (2.07)	2.59 (1.74)	2.29 (1.70)	0.385	0.897*	1.085*	0.481	0.663*	0.175
Describing^a^ *M* (*SD*)	9.93 (5.48)	6.92 (3.64)	7.16 (4.37)	5.43 (4.26)	0.616*	0.531*	0.902*	−0.061	0.372	0.403
Finding *M* (*SD*)	5.98 (1.92)	5.26 (2.34)	4.19 (2.16)	4.17 (2.22)	0.345	0.902*	0.881*	0.470	0.480*	0.009

*Note.* Pairwise comparisons used Tukey's honestly significant difference test to maintain an experiment-wise Type I error rate of .05 for each model. Asterisks indicate a comparison is significant at the .05 level. TD = typical development; DLD = developmental language disorder; DYS = dyslexia.

a The describing task had a maximum possible accuracy score of 40. The maximum score achieved by participants in the sample was 28. All other word learning tasks had a maximum accuracy score of 8.

In the listening task, which measured phonological recognition, the TD group scored significantly higher than the dyslexia-only (*g* = 0.93) and DLD + dyslexia (*g* = 1.24) groups. Additionally, the DLD-only group scored significantly higher than the DLD + dyslexia group (*g* = 0.74). No other group differences were statistically significant (all other effect sizes were small to moderate, *g* < 0.44).

The same pattern was observed in the drawing task, which measured nonverbal semantic recall. The TD group obtained significantly higher drawing accuracy scores than the dyslexia-only (*g* = 0.91) and DLD + dyslexia (*g* = 1.09) groups, and the DLD-only group also scored significantly higher than the DLD + dyslexia group (*g* = 0.66). No other group differences were statistically significant (all other effect sizes were small to moderate, *g* < 0.50).

In the describing task, which measured verbal semantic recall, the TD group obtained significantly higher describing accuracy scores than all other groups: DLD-only (*g* = 0.62), dyslexia-only (*g* = 0.55), and DLD + dyslexia (*g* = 0.90). No other group differences were statistically significant (all other effect sizes were small, *g* < 0.39).

In the finding task, which measured semantic recognition, the TD group scored significantly higher than the dyslexia-only (*g* = 0.90) and DLD + dyslexia (*g* = 0.88) groups. Also, the DLD-only group scored significantly higher than the DLD + dyslexia group (*g* = 0.48). No other group differences were statistically significant (all other effect sizes were small–moderate; all *g* < 0.47).

In summary, the primary analyses of accuracy scores for the five tasks indicated that the TD group consistently obtained the highest scores. The DLD-only group differed from the TD group on only one task, the describing task, whereas the dyslexia-only and DLD + dyslexia groups differed from the TD group on all five tasks. The DLD-only group scored significantly higher than the DLD + dyslexia group on three tasks, listening, drawing, and finding. The dyslexia-only and DLD + dyslexia group did not differ from each other, and the lowest means for all five tasks were observed in these two groups.

### Group Differences in Secondary Scores

The primary analyses examined group differences in global scores that required correct links between phonological forms and semantic referents. We also conducted secondary analyses to examine the extent to which participants learned additional phonological or semantic information that may have been incorrectly linked. Table 6 provides mean recall scores and difference scores for each group for the naming, drawing, and describing tasks. Recall scores reflect participants' ability to store and recall phonological (naming) and semantic information (drawing and describing) regardless of whether they were correctly linked. Difference scores represent the difference between the recall and accuracy scores. Larger difference scores indicate that participants recalled more information than was reflected in their accuracy scores and may highlight difficulties with linking that may not be apparent from the primary measures of global word learning accuracy.

**Table 6.** Recall and difference scores, *M* (*SD*)*,* of select word learning tasks by group.

	Group scores			
Scoring method	TD (*n* = 90)	DLD (*n* = 53)	DYS (*n* = 32)	DLD + DYS (*n* = 69)
Naming recall	1.70 (1.36)	1.38 (1.32)	0.84 (0.85)	1.07 (1.13)
Naming difference	0.11 (0.41)	0.17 (0.38)	0.19 (0.40)	0.17 (0.38)
Drawing recall	5.57 (1.64)	5.13 (1.96)	5.12 (1.58)	4.17 (1.90)
Drawing difference	1.28 (1.25)	1.60 (1.41)	2.53 (1.68)	1.88 (1.71)
Describing recall	12.38 (4.88)	9.74 (4.25)	11.56 (4.49)	8.54 (4.13)
Describing difference	2.44 (2.81)	2.81 (3.22)	4.41 (4.17)	3.10 (2.83)

*Note.* The difference score was created by subtracting the accuracy score (see Tables 3 and 5) from the recall score.

For the naming task, difference scores were low for all groups, ranging from .11 to .19. These results indicate that when participants produced one of the pseudowords, it was almost always linked with the correct object referent. Participants rarely produced correct phonological forms for mismatched referents. Because naming difference scores did not meet assumptions for parametric analyses, we did not analyze them further, but minimal group differences were apparent from the descriptive data.

For the drawing task, the mean difference scores for each group ranged from 1.28 to 2.53, indicating that participants in all four groups tended to recall more objects than they were able to link with the correct pseudoword. Drawing difference scores were analyzed with Welch's ANOVA due to a violation of the homogeneity of variance assumption. The drawing difference scores significantly differed by group, *F*(3, 99.14) = 5.79, *p* = .001. Children in the dyslexia-only group had significantly larger difference scores than children in the TD group (*g =* 0*.*91) and the DLD-only group (*g =* 0*.*61). Note that the mean recall score for the dyslexia-only group was more similar to the DLD-only and TD groups' recall scores, although their accuracy scores were quite low. These results demonstrate that, relative to the TD group and the DLD-only group, children with dyslexia-only demonstrated significantly more semantic learning than they were able to convey on the primary accuracy measure that required correct links between phonological and semantic representations. No other group differences were statistically significant.

For the describing task, the mean difference scores for each group ranged from 2.44 to 4.41, indicating that participants in all four groups tended to recall more semantic information than they were able to link with the correct pseudoword. There was a significant effect of group on the describing difference scores, *F*(3, 240) = 3.21, *p* = .024, such that children in the dyslexia-only group had significantly larger difference scores than children in the TD group (*g =* 0*.*61). Again, relative to the TD group, children with dyslexia-only demonstrated significantly more semantic learning than was indicated by the primary accuracy score. No other group comparisons were statistically significant.

Secondary analysis of the listening task focused on the types of errors made by each group. Recall that, in this task, participants were provided four response options, representing the correct answer and three foils: phonological, semantic, or combined. If participants gave a different response from those presented, errors were classified as “other.” We report the proportion of responses by type and group in Table 7. The general pattern of responses was the same across all four groups, such that phonological errors were the most common error type, followed by semantic errors, combined errors, and other errors, respectively. Descriptively, the DLD-only and dyslexia-only groups made a similar proportion of phonological error responses, which was higher than the TD group but lower than the DLD + dyslexia group. These phonological errors suggest that participants may have formed coarse phonological representations that were linked with the correct semantic referent but lacked phonetic detail. However, the dyslexia-only and DLD + dyslexia group made a similar proportion of semantic, combined, and other error responses, which was higher than the TD and DLD-only groups who performed similarly to each other. A higher proportion of semantic and combined errors in the dyslexia-only and DLD + dyslexia groups suggest unstable or incorrect links between the phonological forms and semantic referents.

**Table 7.** Proportion of listening task responses by type and group.

	Group			
Listening task response	TD (*n* = 90)	DLD (*n* = 53)	DYS (*n* = 32)	DLD + DYS (*n* = 69)
Accurate	69%	60%	51%	43%
Phonological error	14%	21%	20%	28%
Semantic error	10%	11%	16%	15%
Combined error	4%	5%	10%	9%
Other error	2%	2%	4%	4%
Total	100%	100%	100%	100%

*Note.* TD = typical development; DLD = developmental language disorder; DYS = dyslexia.

## Discussion

Previous research has suggested that word learning abilities are negatively impacted for children with dyslexia and children with DLD, but studies have rarely considered or accounted for the high co-occurrence of the two disorders in their participant recruitment or data analysis. This study was the first to examine word learning abilities in children with DLD-only, dyslexia-only, co-occurring DLD + dyslexia, and TD. In addition to distinguishing between children with DLD-only and dyslexia-only, other strengths of this study include the relatively large sample size, our recruitment of participants from class-wide language and literacy screens to provide a broader representation of the subgroups than might be obtained from caseloads or clinical referrals, and the consideration of multiple measures of word learning. We conducted analyses for groups classified with and without a minimum nonverbal IQ criterion (see Supplemental Material S1), and findings were consistent across both methods.

Based on assumptions of core phonological deficits in children with dyslexia (Lyon et al., 2003) and broader language deficits including syntax, semantics, and higher level skills in children with DLD (Leonard, 2014), we predicted that children with dyslexia and DLD would demonstrate different word learning strengths and weaknesses. Specifically, we predicted that children with dyslexia would have difficulty learning phonological word forms, showing difficulties primarily with naming and listening tasks, whereas children with DLD would have difficulty with semantic aspects of word learning, showing difficulties with drawing, describing, and finding tasks. Thus, children with DLD + dyslexia should show difficulty across all five tasks. Our predictions were partially supported in that weaknesses of the DLD-only group primarily involved the recall of semantic information, and children with DLD + dyslexia often showed the poorest learning. However, we were surprised by the range and severity of word learning difficulties observed in the dyslexia-only group, which was greater than we predicted and more severe than would be expected based on their existing vocabulary scores.

### Primary Analyses

Analyses comparing the four groups' performance on the primary measures of word learning accuracy indicated that both groups of children with dyslexia (dyslexia-only and DLD + dyslexia) performed significantly worse than the TD group across all five word learning tasks. The effect sizes for comparisons of the TD group versus the dyslexia-only group ranged from moderate (*g* = 0.55–0.70 for the describing and naming tasks) to large (*g* = 0.90–0.93 for all other tasks); effect sizes comparing the TD group to the DLD + dyslexia group also ranged from moderate to large (*g =* 0*.*55–1.24). Such results indicate that the word learning difficulties of children with dyslexia were not restricted to learning phonological word forms, nor to situations where the response modality was verbal (cf. Baron et al., 2018; Litt & Nation, 2014), but instead were observable across all tasks and response modalities.

In contrast to children with dyslexia, the children in the DLD-only group differed significantly from the TD group on only one primary outcome measure, the describing task, which measured verbal semantic recall. Across all tasks, the effect sizes for comparisons of the TD group to the DLD-only group ranged from small (*g* = 0.28–0.39 for three tasks) to moderate (*g* = 0.43–0.62 for the listening and describing tasks). With the exception of the describing task, the effect sizes for the DLD-only versus TD group comparisons in the current study were smaller than the average effect size (.6) obtained in Kan and Windsor's (2010) meta-analysis of word learning in children with language impairment. Presumably, many studies in the meta-analysis would have included children with comorbid DLD + dyslexia.

In the current study, the DLD-only group also performed significantly better than the DLD + dyslexia group on three out of five primary outcomes, with moderate to large effect sizes (*g =* 0*.*48–0.74). Although differences between the DLD-only group and the dyslexia-only group were not statistically significant, the effect sizes were moderate and favored the DLD-only group for four out of five primary outcomes (*g* = 0.41–0.49); there was minimal difference between the DLD-only and dyslexia-only groups on the describing task (*g* = −0.04). Taken together, these results suggest that the word learning difficulties of children with DLD-only were relatively mild, with the largest deficit relative to the TD group observed for the verbal semantic recall task. In contrast, the word learning difficulties of children with dyslexia (both dyslexia-only and DLD + dyslexia) were moderate to severe considering all five primary tasks.

### Secondary Analyses

Because all of the primary outcomes of word learning accuracy required the word form and the referent to be correctly linked, none can be considered a pure measure of phonological versus semantic word learning. Moreover, participants sometimes recalled phonological forms, semantic referents, or semantic features that were not correctly linked. Our secondary analyses examined these responses to better characterize the nature of word learning difficulties for each group by comparing difference scores in the naming, drawing, and describing tasks, which reflected the difference between the number of items that were recalled and the number of items that were recalled and accurately linked, and by comparing the frequency of different error types on the listening task.

#### Phonological Tasks

On the naming task, which is arguably the most demanding of phonological skills, difference scores were equally low across all groups. Phonological recall was limited for all groups, regardless of whether newly learned pseudowords needed to be linked to the correct referent or not. Turning to the listening task, phonological errors were the most common error type for all groups. Taken together with the primary accuracy scores, the results highlight how difficult it was for participants to learn and produce new, precise phonological forms.

#### Semantic Tasks

Participants in all four groups were able to draw more objects and recall more semantic features than they were able to link with the correct pseudoword. However, compared to TD children, this effect was more pronounced for children with dyslexia-only. Descriptively, the dyslexia-only group recalled a similar amount of semantic information as the TD group on both the drawing and describing tasks. However, difference scores (reflecting the difference between recall and accuracy scores) were significantly larger for the dyslexia-only group on both the drawing and describing tasks than the TD group. The dyslexia-only group's difference score for the drawing task was also significantly larger than the difference score for the DLD-only group. These results suggest that the dyslexia-only group had more difficulty linking newly acquired semantic knowledge with the correct pseudoword. In contrast, the DLD-only group's difference scores did not significantly differ from the TD group's, suggesting that forming new links was not an area of relative weakness for the DLD-only group. Similarly, on the listening task, semantic, combined, and other errors were more common among the dyslexia-only and DLD + dyslexia groups than the DLD-only and TD groups. These results are consistent with those of the finding task, in which the dyslexia-only group and DLD + dyslexia group showed similar levels of difficulty linking pseudowords with semantic referents when both were provided, whereas the DLD-only group did not differ from the TD group. Overall, the findings of the secondary analyses add clarity to the primary accuracy analyses, indicating that the word learning difficulties of children with dyslexia extend beyond the formation and recall of new phonological representations and also include the linking of semantic and phonological information.

### Summary and Future Directions

This study is the first to directly compare the word learning abilities of children with separate versus comorbid DLD and dyslexia to each other and TD children. The primary analyses indicated that children with dyslexia (dyslexia-only and DLD + dyslexia) exhibited significant, moderate-to-severe difficulties with multiple aspects of spoken word learning from direct instruction, whereas children with DLD-only had only mild word learning difficulties, involving the recall of verbal semantic features. Secondary analyses provided further evidence that children with dyslexia struggle to correctly link any additional semantic information they might learn with its phonological counterpart, whereas this was not an area of specific weakness for children with DLD-only. These are important findings, as previous studies of word learning in children with DLD have not controlled for comorbid dyslexia. However, this pattern of results is quite paradoxical considering the broader language profiles of the dyslexia-only and DLD-only groups. Recall the dyslexia-only group had oral language skills well within the average range on a comprehensive language assessment. Moreover, their mean scores on the descriptive, norm-referenced measures of receptive and expressive vocabulary size were slightly above the normative mean. In contrast, the DLD-only group scored poorly on the comprehensive language assessment, and their mean scores on descriptive, norm-referenced measures of expressive and receptive vocabulary size were significantly lower than both dyslexia-only and TD groups. Whereas past studies have highlighted the role of phonological memory in new word learning, the dyslexia-only and DLD-only groups did not differ on the CTOPP-2 phonological memory composite in this study. Thus, how is it that children with DLD-only developed poorer overall vocabulary knowledge if their experimental word learning performance was stronger than children with dyslexia-only? Moreover, how did children with dyslexia-only develop normal vocabulary knowledge despite substantial word learning difficulties?

The results of this study call for additional research to understand the surprising pattern of effects. First, replication studies are necessary to determine the extent to which the findings are restricted to the specific word learning paradigms we used in the study. Although difficult to explain, our finding that children with dyslexia-only exhibit word learning deficits in the presence of normal vocabulary size was consistent with those of Alt et al. (2017), who used a different type of instructional paradigm involving guessing with feedback, as well as different measures of word learning. Alt et al. found that children with dyslexia-only exhibited deficits in primarily phonological aspects of word learning, but also in visually complex tasks. However, additional studies are needed that include all four DLD/dyslexia subgroups to confirm the pattern of results shown in this study regarding DLD-only versus TD and dyslexia-only.

Second, similar to many other word learning experiments (e.g., Gray, 2006; Litt & Nation, 2014), the novel word learning instruction provided to children in this study was highly explicit but featured relatively shallow contextual content. Additionally, we incorporated principles from the learning sciences to boost students' learning (cf. Haebig et al., 2019; Leonard et al., 2019; Roediger & Butler, 2011). Children were given multiple exposures to the word–object pairs, and we incorporated spaced retrieval practice to help facilitate their learning of phonological and semantic information. This type of instruction differs from “natural” word learning, where most words are learned incidentally via experience in meaningful communicative contexts. It may be that children with DLD-only respond better to explicit, systematic vocabulary instruction of the type used in this study, but children with dyslexia-only are better equipped for word learning in natural contexts. Additional research should explore this hypothesis; if correct, it would provide important information about approaches to vocabulary intervention for children with DLD versus dyslexia. Indeed, Haebig et al. (2019) found that spaced retrieval practice mitigated some word learning differences between preschool children with DLD (whose dyslexia status was unknown) and TD peers.

Third, in this study, word learning was measured at a single time point, immediately after the explicit instruction was provided. Future studies should examine to what extent the newly acquired knowledge is retained over time. Studies including delayed posttests of word learning (e.g., 6–21 days after instruction) often note a decline in remembered information (Ricketts et al., 2008; Storkel et al., 2019, but see Leonard et al., 2019). It is possible that the DLD and dyslexia subgroups differ in their long-term retention of learned information, and this could lead to different results for experimental word learning tasks versus measures of overall vocabulary knowledge. This is another direction for future research that could provide important information about how to support vocabulary growth in individuals with different types of language-based learning disabilities.

## Conclusions

We examined spoken word learning abilities in children with DLD-only, dyslexia-only, DLD + dyslexia, and TD. Results indicated that, in response to explicit oral instruction, children with dyslexia, including those in the dyslexia-only and DLD + dyslexia groups, showed significantly poorer word learning than their TD peers across multiple tasks measuring phonological and semantic aspects of word learning. Surprisingly, the DLD-only group showed relatively mild word learning deficits and only differed from TD peers on a task measuring verbal semantic recall of learned words. Overall, the results of this study highlight the value of studying DLD and dyslexia subgroups separately. Prior studies have suggested both disorders are associated with word learning difficulties but have not accounted for their frequent co-occurrence in their sampling or analysis procedures. More studies are needed to understand why children with DLD show delayed vocabulary learning, how some children with dyslexia develop strong vocabularies despite significant word learning difficulties, and how best to close vocabulary gaps to promote strong reading and other academic skills in children with language-based learning disabilities.

## Author Contribution

**Suzanne M. Adlof:** Conceptualization (Lead), Funding Acquisition (Lead), Supervision (Lead), Formal analysis (Support), Writing – original draft (Support), Writing – review & editing (Lead). **Lauren S. Baron:** Formal analysis (Support), Writing – original draft (Support), Writing – review & editing (Support). **Bethany A. Bell:** Formal analysis (Lead), Writing – original draft (Support). **Joanna Scoggins:** Data curation (Lead), Investigation (Lead).

## Supplementary Material

10.1044/2021_JSLHR-20-00217SMS1 Supplemental Material S1 Analyses using more restrictive inclusionary criteria, as is common in studies of specific language impairment (SLI; i.e., nonverbal intelligence scores > 85). The document contains three tables: A. Descriptive statistics and group comparisons for subgrouping and descriptive assessments using SLI criteria; B. One-way ANOVA summary table examining group differences for the primary outcome for each word learning task using SLI criteria; and C. Primary accuracy scores of word learning tasks by group using SLI criteria; Effect size and significance of pairwise comparisons.

10.1044/2021_JSLHR-20-00217SMS2 Supplemental Material S2 Additional information about the word learning training script.
