# Supplementary material for: Spoken Word Learning in Children With Developmental Language Disorder or Dyslexia
Source: J Speech Lang Hear Res. 2021 Jun 28;64(7):2734–49. doi: 10.1044/2021_JSLHR-20-00217 (PMC8632516; doi:10.1044/2021_JSLHR-20-00217)
Supplement: Supplemental Material S1 [file JSLHR-64-2734-s001.pdf]

Supplemental Material S1

A. Descriptive statistics and group comparisons for subgrouping and descriptive assessments using SLI criteria.

	Group Scores				ANOVA Results				
**Subgrouping Assessment**	**TD (*n* = 86)**	**SLI (*n* = 47)**	**DYS (*n* = 30)**	**SLI+DYS (*n* = 60)**	***df* (3,**	***F***	***p***	**R^2**	**Group Comparison**
CELF-4 *M (SD)*	101.01 (6.99)	79.47 (5.13)	97.47 (5.67)	73.82 (8.68)	219	221.82	< .001	.752	TD = DYS > SLI > SLI+DYS
WRMT-3 *M (SD)*	101.23 (6.11)	101.53 (7.75)	80.23 (3.70)	74.82 (7.40)	219	258.69	< .001	.777	TD = SLI > DYS > SLI+DYS
TONI-4 *M (SD)*	106.73 (8.68)	103.57 (6.94)	102.30 (7.76)	96.92 (7.29)	219	18.62	< .001	.203	TD = SLI > SLI+DYS, SLI = DYS, TD > DYS > SLI+DYS
**Descriptive Assessment**	**TD [a]**	**SLI**	**DYS**	**SLI+DYS [b]**	***df* (3,**	***F***	***p***	**R^2**	**Group Comparison**
TOWRE-2 *M (SD)*	102.15 (9.77)	100.77 (9.76)	79.00 (8.17)	74.93 (12.03)	219	111.09	< .001	.603	TD = SLI > DYS = SLI+DYS
CTOPP-2 *M (SD)*	92.64 (11.25)	83.15 (10.25)	88.50 (12.47)	75.98 (9.61)	218	29.27	< .001	.287	TD = DYS > SLI+DYS, DYS = SLI, TD > SLI > SLI+DYS
PPVT-4 *M (SD)*	106.64 (10.77)	94.13 (8.042)	104.87 (8.76)	91.76 (9.02)	217	37.02	< .001	.339	TD = DYS > SLI = SLI+DYS
EVT-2 *M (SD)*	105.88 (8.71)	93.17 (7.29)	101.07 (6.19)	87.75 (8.55)	218	65.90	< .001	.476	TD > DYS > SLI > SLI+DYS

[a] Sample size differed on the PPVT-4 (*n* = 85).
[b] Sample sizes differed on the CTOPP-2, PPVT-4, and EVT-2 (*n* = 59).
Note. A series of one-way ANOVAs were estimated to examine group differences on the subgrouping and descriptive assessments. Pairwise comparisons using Tukey's HSD were applied to maintain an experiment-wise Type I error rate of .05 for each model.

B. One-way ANOVA summary table examining group differences for the primary outcome for each word learning task using SLI criteria.

Naming

Source	Sum of Squares	*df*	Mean Square	*F*	*p*-value	R^2
Group	25.00	3	8.33	5.61	.001	.071
Error	325.18	219	1.49			

Listening

Source	Sum of Squares	*df*	Mean Square	*F*	*p*-value	R^2
Group	179.57	3	59.86	22.07	< .0001	.232
Error	593.93	219	2.71			

Drawing

Source	Sum of Squares	*df*	Mean Square	*F*	*p*-value	R^2
Group	181.07	3	60.36	16.98	< .0001	.189
Error	778.37	219	3.55			

Describing*

Source	Sum of Squares	*df*	Mean Square	*F*	*p*-value	R^2
Group	795.41	3	265.14	11.35	< .0001	.146
Error	4642.34	98.6	21.20			

Finding

Source	Sum of Squares	*df*	Mean Square	*F*	*p*-value	R^2
Group	161.70	3	53.90	12.19	< .0001	.143
Error	968.28	219	4.42			

*Results are from Welch’s ANOVA to account for unequal group variances.

C. Primary accuracy scores of word learning tasks by group using SLI criteria; Effect size and significance of pairwise comparisons.

	**Group Descriptives**				**Effect Size (Hedge's *g*) and Significance of Pairwise Comparisons**					
**Task**	**TD (*n* = 86)**	**SLI (*n* = 47)**	**DYS (*n* = 30)**	**SLI+DYS (*n* = 60)**	**TD vs. SLI**	**TD vs. DYS**	**TD vs. SLI+DYS**	**SLI vs. DYS**	**SLI vs. SLI+DYS**	**DYS vs. SLI+DYS**
Naming										
M (SD)	1.53 (1.35)	1.17 (1.36)	0.63 (0.72)	0.88 (1.09)	0.266	0.737 *	0.517 *	0.468	0.236	-0.258
Listening										
M (SD)	5.47 (1.56)	4.74 (1.81)	4.07 (1.48)	3.27 (1.71)	0.442	0.908 *	1.359 *	0.396	0.841 *	0.491
Drawing										
M (SD)	4.26 (1.97)	3.64 (2.07)	2.63 (1.79)	2.12 (1.65)	0.309	0.846 *	1.162 *	0.514	0.826 *	0.303
Describing										
M (SD)	9.70 (5.44)	6.89 (3.66)	7.00 (4.23)	5.07 (4.10)	0.575 *	0.523 *	0.939 *	-0.028	0.466	0.466
Finding										
M (SD)	5.90 (1.92)	5.23 (2.33)	4.07 (2.16)	3.97 (2.13)	0.323	0.922 *	0.962 *	0.512	0.569 *	0.048

Note. Pairwise comparisons used Tukey’s HSD to maintain an experiment-wise Type I error rate of .05 for each model. Asterisks indicate a comparison is significant at the .05 level. TD = typical development, SLI = Specific Language Impairment, DYS = Dyslexia.
