# Supplementary material for: Spoken Word Learning in Children With Developmental Language Disorder or Dyslexia
Source: J Speech Lang Hear Res. 2021 Jun 28;64(7):2734–49. doi: 10.1044/2021_JSLHR-20-00217 (PMC8632516; doi:10.1044/2021_JSLHR-20-00217)
Supplement: Supplemental Material S2 [file JSLHR-64-2734-s002.pdf]

**Supplemental Material S2.** Structure of word-learning training script.

Children were instructed that they would be helping an astronaut learn about life on an alien planet before she traveled there. Within each of three teaching blocks, children were taught the name for each object referent, its semantic category, and four semantic features. They also had one opportunity to name the object (phonological recall) and one opportunity to find the object within an array of all objects (semantic recognition), and they received recorded feedback on the accuracy of their naming and finding responses. The order of the script elements was varied within and across the three teaching blocks offering retrieval practice opportunities. Within each teaching block, the order of presentation of the target words was randomized. Across the three blocks, children received 22 exposures to the spoken word form, three phonological recall opportunities and three semantic recall opportunities with feedback. After each block, children received "space coins" to spend in the "space store" as positive reinforcement.

| **Script Framework** | **Script for Item 1** 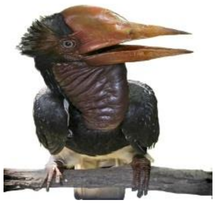 |
|---|---|
| *Training Block 1* | |
| This is a [word].<br>A [word] is a type of [category].<br>A [word] [feature 1].<br>It [feature 2].<br>A [word] [feature 3].<br>A [word] [feature 4].<br>Can you find the [word]?<br>Yes, that's the [word]!*<br>What's this called? You say it.<br>Right! It's a [word].** | This is a /felɪdʒ/.<br>A /felɪdʒ/ is a type of bird.<br>A /felɪdʒ/ has wrinkled brown skin on its neck.<br>It has a large bony plate on its beak.<br>A /felɪdʒ/ eats bugs.<br>A /felɪdʒ/ likes warm weather.<br>Can you find the /felɪdʒ/?<br>Yes, that's the /felɪdʒ/!<br>What's this called? You say it.<br>Right! It's a /felɪdʒ/. |
| *Block 1 Reinforcement* | |
| *Training Block 2a* | |
| Do you know what this is called?<br>Right! It's a [word].**<br>A [word] is a type of [category].<br>A [word] [feature 1].<br>A [word] [feature 3]. | Do you know what this is called?<br>Right! It's a /felɪdʒ/.<br>A /felɪdʒ/ is a type of bird.<br>A /felɪdʒ/ has wrinkled brown skin on its neck.<br>A /felɪdʒ/ eats bugs. |
| *Training Block 2b* | |
| Can you find the [word]?<br>Yes, that's the [word]!*<br>It [feature 2].<br>A [word] [feature 4]. | Can you find the /felɪdʒ/?<br>Yes, that's the /felɪdʒ/!<br>It has a large bony plate on its beak.<br>A /felɪdʒ/ likes warm weather. |
| *Block 2 Reinforcement* | |

| | |
|---|---|
| *Training Block 3a* | |
| Can you find the [word]?<br>Yes, that's the [word]!*<br>A [word] [feature 1].<br>A [word] [feature 3]. | Can you find the /felɪdʒ/?<br>Yes, that's the /felɪdʒ/!<br>A /felɪdʒ/ has wrinkled brown skin on its neck.<br>A /felɪdʒ/ eats bugs. |
| *Training Block 3b* | |
| Do you know what this is called?<br>Right! It's a [word].**<br>A [word] is a type of [category].<br>It [feature 2].<br>A [word] [feature 4]. | Do you know what this is called?<br>Right! It's a /felɪdʒ/.<br>A /felɪdʒ/ is a type of bird.<br>It has a large bony plate on its beak.<br>A /felɪdʒ/ likes warm weather. |
| *Final Reinforcement* | |

* Correct response feedback. The feedback for an incorrect response was "Not quite. Here's the [word]."

** Correct response feedback. The feedback for an incorrect response was "It's a [word]."
